# Multiple Sclerosis and Schizophrenia

**DOI:** 10.3390/ijms18081760

**Published:** 2017-08-12

**Authors:** Borros M. Arneth

**Affiliations:** Institute of Laboratory Medicine and Pathobiochemistry, Molecular Diagnostics, Hospital of the Universities of Giessen and Marburg (UKGM), Justus Liebig University Giessen, Feulgenstr. 12, 35392 Giessen, Germany; borros.arneth@klinchemie.med.uni-giessen.de; Tel.: +49-641-985-41556

**Keywords:** schizophrenia, multiple sclerosis, autoimmune diseases, autoantibodies, anti-myelin antibodies, anti-glutamate receptor antibodies

## Abstract

The psychiatric and neurological aspects of health may present methodological challenges in the diagnosis and treatment of disease. This is especially true for patients whose symptoms indicate the coexistence of multiple sclerosis (MS) and schizophrenia (SCZ). These cases raise critical questions regarding the relationship between the mind and the brain. Studies have noted that patients with MS have an increased risk of developing SCZ or bipolar disorder (BD). It is suggested here that MS and a subgroup of SCZ have similar etiologies. Factors such as gender, ethnicity, geography and season also have an influence on the occurrence of MS and SCZ. This paper aims to examine the differences and similarities between SCZ and MS. For this purpose, scientific papers examining various factors associated with these disorders were reviewed, and similarities and differences in genetic, immunological, seasonal, geographical, and gender-related risk factors and limited similarities in ethnic factors between the two diseases were identified. The findings suggest that subgroups of these two diseases may belong to the same class of disorders.

## 1. Introduction

Schizophrenia (SCZ) is a severe mental disorder that is among the leading causes of disability worldwide [1] and has major effects on the quality of life of individuals who are directly or indirectly affected. Multiple sclerosis (MS), a disease that affects the central nervous system [1], has a similar impact on the quality of life. According to several studies, the inflammatory process that occurs in MS patients is directly associated with human leukocyte antigen (*HLA*) class I and II loci [2,3]. SCZ and MS both impose substantial costs on society. The underlying pathological mechanisms and genetic risk factors associated with these disorders remain unclear. Despite the distinct nature of these two disorders, we have identified characteristics that are shared between MS and SCZ. Various research studies have identified risk factors, such as age of onset, seasonal variations in birth date and time of disease onset, and immunological factors, as well as differences in gender and ethnic distribution, that are associated with both SCZ and MS. This paper seeks to compare these factors in connection with SCZ and MS and to identify the similarities and differences between them.

Subgroups of MS and SCZ may belong to the same class of disorders due to similarities in the age of onset, geographical distribution and immunological response of patients with the diseases [1]. MS is defined as a chronic disease that affects the central nervous system [4], whereas SCZ is defined as a primarily psychiatric disease [5,6]. These diseases have both been extensively investigated as a result of the increased interest they have attracted in the scientific realm. From this perspective, the purpose of this review is to discuss the similarities and differences between MS and SCZ.

As a psychiatric disease, SCZ is recognized as a disorder that involves an interplay between environmental and genetic factors [5]. Several studies have indicated that immune challenges can contribute to mental disorders. Multiple studies have also examined the effects of genetic and antigenic exposure [7] on SCZ and MS. Fetal and early childhood immune challenges seem to increase the risk of developing a psychiatric disorder [7].

Subgroups of MS and SCZ share several similarities. For example, although these disorders are clinically and pathologically distinct, both can be triggered by infections and tend to begin in early adult life.

## 2. Onset and Causal Factors

The two diseases have numerous similarities in terms of the onset and cause. SCZ typically first manifests in young adults, with an age distribution of 15–45 years [8]. SCZ affects the same age distribution as MS; however, it has a 10- to 100-fold higher estimated prevalence rate [1]. The onset of both diseases at the acute or subacute level is characterized by a progressive disability or periods of remission and exacerbation (acute episodes) [1]. Both illnesses tend to lack clinical signs and symptoms; however, easy fatigability is common in the early stages of MS, and low-grade headaches are common in the early stages of SCZ [1].

At clinical onset, more than 85% of MS patients are classified as having relapsing remitting MS (RRMS). These patients are predominately female and typically 20–30 years old at the presentation of initial symptoms. The remaining 10–15% of MS patients exhibit primary progressive MS (PPMS), which is characterized by a continuous worsening of symptoms from the onset, when patients are typically between 30 and 50 years old [4,5].

## 3. Geographical Factors

The geographic distributions of SCZ and MS have long been the subject of debate among scientists. For example, SCZ has similar prevalence rates worldwide [9]. The different diagnostic criteria for SCZ in various parts of the world, especially in developing countries, have significantly affected epidemiological studies. Data reported primarily from Africa are based on hospital incidence rates rather than surveys [9]. These reports are not reliable because of the small number of medical facilities and communities’ considerable tolerance for disease [9]. These reported incidence rates, however low, are erroneously high in many developing countries as a result of misdiagnosis [9].

A study conducted between 1983 and 1984 in Harare, Zimbabwe indicated that of all patients admitted to two university-affiliated hospitals, 40–50% were diagnosed with SCZ [9]. When the Research Diagnostic Criteria [10] were applied, more than half of the patients failed to meet the criteria and were diagnosed with brief reactive psychosis. Moreover, when DSM-III-R criteria were used, only 10–15% of the patients met the criteria for a SCZ diagnosis. Thus, the available data cannot support the indication that SCZ is equally prevalent worldwide. In addition, MS is substantially more widespread in the northern and temperate regions of the world than in the tropics [9]. Similarly, reported cases of SCZ are more prevalent in Northern and Western Europe and less common in Southern Europe and Africa [9].

The minimum set duration to diagnose SCZ may result in incorrect data regarding the incidence rate. In the USA, the number of admitted patients diagnosed with SCZ throughout the country exhibits a significant geographical difference from the corresponding number for MS. This difference arises because the USA does not have a minimum set duration for SCZ diagnosis; thus, data sets may include patients with brief psychosis [1]. Finally, studies have concluded that SCZ occurs at equal rates worldwide because the data set used in these studies consisted of incidence data derived from the Present State Examination CATEGO (PSE-CATEGO) definition of SCZ [1]. Although the PSE-CATEGO definition of SCZ is highly effective, it does not differentiate SCZ from many acute psychotic disorders [11] and thus substantially compromises the validity of the data. For the sake of accuracy, new SCZ epidemiology studies should take into account the diagnostic criteria.

The hypothesis that environmental factors and genetic predisposition explain the epidemiology of SCZ, as in MS, conforms to the unequal geographical distribution, which includes pockets of very high incidence [1]. The available data on SCZ indicate that the hypothesis that SCZ is equally distributed worldwide is premature. In developing countries, MS has a poorer prognosis than SCZ, which has a slightly better prognosis in the tropics than in temperate lands [9].

The better SCZ prognosis in developing countries may be a result of an exaggerated incidence rate caused by the inclusion of brief psychotic disorders in the category of SCZ. Another factor that may contribute to the better prognosis in developing countries might be increased immunity to SCZ-inducing agents (as infections) [9]. Moreover, success rates may be attributed to the extensive use of electroconvulsive therapy over prolonged neuroleptic treatment in the developing world [9].

The migration of people from high-risk to low-risk environments and vice versa is another factor that shows up the similarities and differences between MS and SCZ. Adults who migrate from relatively high-risk countries to low-risk regions have the same chance of being diagnosed with MS as their counterparts who remain in high-risk countries [9]. However, recent data indicate that children who live in low-risk areas until the age of 15 have the same risk of MS as the children they grew up with even if they migrate to a high-risk area [9]. These data have led to the conclusion that shared exposure to the responsible agent at puberty or during childhood may be the cause of the consistent risk rate in the two groups of children. Evidence from Israeli and Scandinavian immigrants who moved to North America indicates that children born in the native country carry the same risk as their parents [12].

Unfortunately, the effects of migration and age on the risk of SCZ have not been adequately investigated. The available data, which are not precisely gathered, indicate that immigrants from high-risk regions continue to carry a high risk even after moving [13]. Other data have shown that immigrants from Africa, India and the Caribbean who move to England are at an increased risk of being diagnosed with SCZ [13].

## 4. Epidemic Factors

Another similarity between MS and SCZ is the epidemic nature of these diseases. Epidemiology addresses the prevalence, distribution, and control of disorders. Prevalence is an estimate of the number of individuals diagnosed with MS or SCZ in a given period. Incidence, on the other hand, estimates the number of individuals newly diagnosed with MS or SCZ in one year. There have been reports of spatial and temporal clusters of increased prevalence. For example, evidence has documented the appearance of MS in the Faeroe Islands after British occupation during World War II. These islands were previously free from MS [14].

A survey in Micronesia yielded similar findings for SCZ [15]. A study conducted in Ghana in the 1950s indicated that of 5000 individuals, only one individual had SCZ. When the same survey was conducted 26 years later, 13 cases of SCZ per 5000 individuals were identified [16]. By the time of the second survey, the population had been exposed to Western civilization. Nevertheless, studies show that there are more individuals affected by SCZ than MS. Currently, the prevalence rate of MS in the USA is between 57 and 78 per 100,000 individuals in the Southern states and 110 to 140 cases per 100,000 in the Northern states, which indicates that approximately 400,000 individuals are affected in the USA and 2.5 million worldwide [2]. Approximately 2.2 million individuals in the USA are affected by SCZ [17]. Both disorders affect young adults. MS commonly manifests in youths between 15 and 20 years of age, whereas SCZ is present mainly in adults older than 18 years of age.

## 5. Genetic Factors Associated with SCZ and MS

Genetic findings show that SCZ and MS share several common risk factors. Many research studies have focused on the significance of genetic risk factors in SCZ; however, few studies have examined genetic predisposition to MS. Nevertheless, the genetic architecture that underlies SCZ and MS susceptibility is similar in some cases. For example, twins have exhibited similar levels of risk for the disorders [3,5]. Monozygotic twins have consistently shown high concordance rates for both MS and SCZ. These concordance rates in identical twins range from 30 to 80% for MS and approximately 50 to 60% for SCZ [18]. Moreover, dizygotic twins exhibit approximately 5–10% concordance rate only. The genetic approach to understanding the causal factors of these disorders may aid in the development of various treatment and prevention regimens. Many more individuals are affected by SCZ than MS. For this reason, the heritability of MS is nearly the same as that of SCZ [19]. Researchers state that MS is not hereditary; however, the presence of an affected sibling increases the risk of the disease. Similarly, parents and children show the same degree of risk concordance as siblings for both MS and SCZ [19,20].

Genome-wide association studies (GWASs) have indicated a significant overlap in genes between SCZ and MS. The studies also identified 21 independent loci associated with SCZ that were also associated with MS [2]. The major histocompatibility complex (MHC) is responsible for the genetic overlap in both MS and SCZ. A GWAS noted the involvement of similar *HLA* alleles in MS and SCZ [2]. Thus, several risk alleles for MS are associated with a decreased risk for SCZ [2].

A study conducted by Picchioni et al. showed a significant association between *HLA* factors and MS in a small subset of the study population [18]. Similarly, other studies have indicated a predominance of *HLA* factors in SCZ patients. In contrast, the same GWAS indicated there was no genetic overlap between bipolar disorder (BD) and MS [2].

Mutations in specific genes are associated with high MS rates, particularly in families in which more than one individual is affected. Regarding SCZ, an individual with an affected first-degree relative has higher chances of developing SCZ than an individual with an affected third-degree relative. Maternal infection by *B. burgdorferi* is also discussed and cannot be excluded. Genetic and antigenic exposure at conception and birth should be emphasized.

For more clarity, the MHC associations identified in the GWAS and other important similarities and differences between MS and SCZ are summarized in Table 1.

## 6. Immunological Factors

A decrease in T-cells, intrathecal IgG, and oligoclonal bands in cerebrospinal fluid during exacerbations of MS are the strongest evidence that MS is an immunological disorder [21].

Reports have stated that cerebrospinal fluid (CSF) IgG and oligoclonal bands increased in 36% of patients diagnosed with SCZ [22]. Another report indicated that T lymphocyte levels are reduced in patients with acute SCZ but are restored to normal in treated patients [23] indicating that a subform or subgroup of SCZ is also caused by immune dysfunction.

A different study of SCZ indicated no reduction in T-cells in chronically hospitalized patients [24]. Most of these patients were on neuroleptics; however, no decrease was identified in natural killer cells [24]. The same finding was reported for MS [25]. However, antibody titers to measles were inconsistently reported in SCZ [26] and MS [27]. Abnormal lymphocytes were also reported in the peripheral blood of SCZ patients [28]. Abnormalities in the lymphocyte nuclei, as well as other lymphocytic and serological abnormalities, including abnormal protein in the CFS and increased “anti-brain” antibodies, have also been reported [1].

Many tests have been conducted to search for numerous viruses and antibodies in the serum and CSF in both MS and SCZ patients. Increased serum titers against influenza A, cytomegalovirus, measles and herpes simplex virus have been reported for SCZ [29]. Other peripheral lymphocytic and serological abnormalities, as well as “ultrastructural abnormalities” in lymphocytes, have been reported for SCZ [28]. In an experiment on monkeys, the IgG component of SCZ serum was able to induce behavioral changes by targeting the septal region of the brain [22]. Similar reactions to SCZ serum have been identified in several other studies [22].

The measles-rubella-varicella zoster (MRZ) reaction, present in many MS patients, is an intrathecal, polyspecific humoral immune response directed against the three most frequent neurotropic viruses: measles (M), rubella (R) and varicella zoster (Z). The reaction is assessed using the three respective antibody indices (AIs). A positive AI typically indicates intrathecal synthesis of antibodies against the corresponding pathogen, which is or recently was present in the patient’s CSF.

The simultaneous absence of virus DNA in the CSF of MS patients with positive MRZ-AI led to the hypothesis of a ‘bystander reaction’, described as polyspecific B cell activation within the CNS in patients with MS. General immune activation is therefore frequently present in the brain tissue of patient with MS.

## 7. Season of Birth

Several studies have established that individuals who develop SCZ in the later stages of their lives tend to be born in winter or at the beginning of spring [30]. Most of these studies attribute these seasonality outcomes with infections that may arise from the cold weather [31,32]. A survey conducted in the English–Welsh population showed that approximately 8% and 4% above chance level of future schizophrenic patients were born in winter and spring months, respectively [33].

Templer et al. (1992) reported results contradicting these studies [30]. Specifically, they did not identify a significant correlation between season of birth and occurrence of SCZ; however, they identified a significant correlation between season and occurrence of MS [30]. Additional controversial results regarding MS and seasonality have been obtained by a study conducted in the Netherlands that indicated individuals born in February and March had an elevated incidence rate of optic neuritis [34]. The interest in the relationship between season of birth and disorder occurrence arises from the possibility of seasonal exposure to risks during conception, birth or special periods of intrauterine growth.

The annual peaks in MS patient birth dates in Denmark and SCZ patient birth dates in Finland are exactly nine months apart [34]. The activity of endemic *Ixodes ricinus* at the time of conception and parturition reflects the seasonal patterns. Similarly, there is a nine-month shift between the annual peaks in MS births in Vancouver and SCZ births in the USA. The periodicity of *Ixodes* tick activity in Europe is exactly mirrored by the seasonal distribution of MS and SCZ [33].

## 8. Season of Onset

The aim of examining the season of onset is to determine the infections and toxic syndromes that may play a role in the incidence of the disorders. In an analysis of mental admissions in both England and Wales, Hare (1983) indicated that the months of July and August had an excess of 9.5% in admissions for SCZ compared with other months [33].

Similar to these findings, the results of a study in Japan showed an increase of approximately 9% inpatient admissions for SCZ within the same period [35]. One study indicated that MS is associated with measles and upper respiratory infections, and frequent attacks of MS occurred during the summer period in Arizona [36].

## 9. Gender Differences in the Occurrence of MS and SCZ

Many medical diseases differentially affect the sexes. Some researchers attribute the differences in the diagnosis time and age of onset of MS to a disproportionate incidence rate in women. The factors that have been identified to skew the risk of SCZ toward women include smoking [37] and the use of oral contraceptives. However, contradictory results have been noted concerning the use of contraceptives and MS.

MS: A study conducted in Canada showed that contraceptives were not associated with an increased number of women with MS [38]. Monozygotic female twins had a higher chance of acquiring MS than their dizygotic twin counterparts [18,20].

SCZ: The results of a study conducted by Seeman showed that men with SCZ had an earlier onset and a less favorable response to treatment than women [39]. Early-onset SCZ predominately affected men, whereas late onset predominantly occurred among women. Regarding treatment, men responded better to treatment without the use of neuroleptics, whereas women responded positively to neuroleptic treatment.

Furthermore, women possessed protective factors that delayed the development of SCZ compared with the typical age of onset for men.

In MS, the difference in gender was a result of changes in women’s lifestyles, including changing gender roles that increased the presence of women in the workforce, different dietary habits, changes in the timing of childbearing and alterations in menarche.

## 10. Ethnic Differences

SCZ: Only a few studies have addressed ethnic differences in the rates of psychiatric diagnosis. Among the studies that have examined ethnicity, many studies have focused on the white and African-American populations, reporting that black patients with SCZ are less symptomatic than white patients [40].

The white population exhibited more hallucinations than the African American population. Moreover, patients who did not originate from the Western world showed few Schneiderian first-rank symptoms [41].

Experts have determined that the perception of emotion in SCZ is relevant in the process of identifying SCZ. A study that aimed to assess the perception of emotion in SCZ showed that African Americans and Latinos had lower scores than their Caucasian counterparts. The results indicate the existence of cultural influences that affect the process of SCZ identification.

MS: Studies have also detected ethnic differences in MS risk factors. One study that aimed to determine ethnic variation in MS incidence showed that Hispanics, Asians, and whites had lowered risks of MS than blacks [42].

Another study showed that African Americans are more likely to experience later onsets of both diseases [42]. In contrast, Hispanic Americans experience disease onset at an earlier age. The average onset age differs across various ethnic patients; however, the length of diagnostic delay is consistent in most studies conducted [42].

The disease course and presentation also vary across ethnic populations in most surveys. African Americans may have transverse myelitis, frequent relapses, and post-relapse recoveries as a result of a more aggressive course. In another study, more elevated CSF IgG was identified in African-American MS patients compared with Caucasian patients [42]. The elevated CFS IgG negatively correlates with gray matter in African Americans diagnosed with MS. These observations raise many possibilities and warrant further investigations.

Limited studies have compared Hispanic-American and Caucasian-American MS patients; however, some research has demonstrated that Hispanic Americans have a higher frequency of transverse myelitis at presentation and optic neuritis than Caucasian Americans [41], which may be a result of their Asian ancestry.

Despite these observations, female preponderance and disease progression are reported to be the same among Hispanic Americans and Caucasian Americans [41].

## 11. Overlap

MS and SCZ may be present together in the same patient. In a recent review, 91 cases were identified in the literature in which both MS and psychotic disorders or mood disorders with psychotic features were present in the same patient [43]. In most cases (> 60%), frontotemporal lesions were present and, in 26 cases, corticosteroids were successfully used for therapy [43].

## 12. Strategies for Further Studies—Family Association Studies

Family investigation studies may be undertaken to investigate the connection between SCZ and MS in more detail; this research should address whether SCZ and MS may be diagnosed in members of the same families. To date, no such studies are available in the literature.

## 13. Pathophysiology

To date, it appears that the pathophysiologies of MS and SCZ are similar but not identical. For MS, the pathophysiology appears to lie in an autoimmune reaction directed against the myelin sheaths of the nerves. Thus, the transmission of information is disrupted. Similarly, novel findings suggest that, at least in a subgroup of patients with SCZ, autoantibodies cause the disease [44]. However, this time, these auto-antibodies appear to be directed against receptors on the perikarya of the nerve cells (NMDA receptors, [44]). This finding may explain the different symptoms and clinical manifestations of the two diseases.

Whether these two diseases are caused by the same or a similar reason or condition remains unclear. The *HLA* genes involved appear to be the same; however, alleles or mutations within these genes appear to have opposite effects in MS and SCZ [5]. *HLA* alleles that promote MS are associated with a reduced risk of the development of SCZ, and vice versa [5]. This finding suggests that there are several questions that remain unresolved.

**Figure 1:**
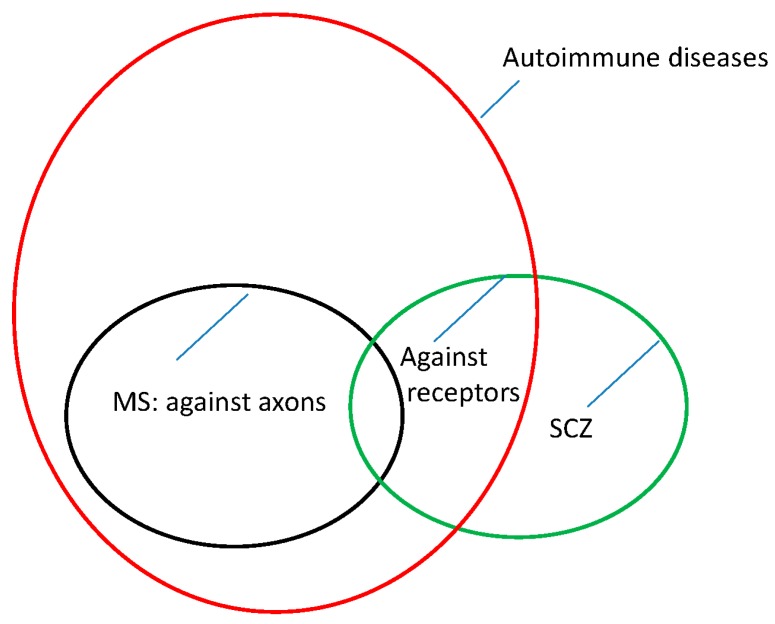
A theoretical picture of the situation: All MS and a subgroup of SCZ cases are thought to be caused by an autoimmune process.

This leads us to visualize the epidemiology and pathophysiology of MS and SCZ in the form of Figure 1.

## 14. Discussion

The literature contains reports of several patients who have been diagnosed simultaneously with SCZ and MS [43]. In general, patients with MS frequently also develop psychiatric problems.

Therefore, it is not clear whether this comorbidity occurs because the MS diagnosis causes mental stress or whether the psyche is attacked by MS.

Regardless, several individuals have been diagnosed with both diseases, MS and SCZ, simultaneously [43].

To date, whether SCZ and MS are often diagnosed in members of the same families has not been adequately investigated and remains an important question for future studies to address.

There may be hereditary elements that predispose patients to developing not only MS or SCZ separately but also both diseases simultaneously, as both diseases can occur in the same family. This would be an interesting point for future studies to investigate.

Specific HLA haplotypes associated with MS and SCZ have been identified (for details, please refer to Table 1).

MS is associated with distinct pathophysiological alterations in the brain and spinal cord, which may be explained by the nature of MS as an autoimmune disease directed against the myelin sheaths of the nerve fibers of the central nervous system.

However, in recent years, a subpopulation of SCZ patients has been identified as having autoantibodies directed against cerebral glutamate receptors [44].

In this context, this finding is interesting because it represents an autoimmune genesis, similar to that of MS. However, autoantibodies against receptors are different from autoantibodies against the myelin sheath, and the resulting clinical manifestations are different as well.

Most symptoms of MS result from the loss of neurological functions, including sensory and/or motoric functions, whereas SCZ frequently manifests as a result of psychiatric disturbances, including changes in affect, awareness and consciousness.

In SCZ, patients frequently cannot distinguish between their imaginations and reality, which may be caused, at least in some cases, by autoantibodies that are directed against cerebral neural receptors and which might stimulate these receptors without adequate reality-derived input.

## 15. Conclusions

MS and SCZ have significant similarities and differences. Their etiological similarities suggest that both diseases may be triggered by similar infectious stimuli.

Neurologists, however, have yet to adopt this concept as widely for SCZ as for MS. There are important differences between MS and SCZ: one difference is that MS is caused by immunologically driven neuropathological changes in the brain and spinal cord.

Another difference is that MS is more prevalent in females than males, whereas the incidence of SCZ is equal in males and females. A further demographic difference is that SCZ is more common in Blacks than Whites, whereas MS affects more whites than blacks. Studies that have examined the prevalence of SCZ and MS may vary in their population sizes, ethnic structures, and age structures, as well as the methods used to quantify disease statistics. This variability limits the quality of comparisons that may be drawn across studies.

## Figures and Tables

**Table 1:** The most important differences and similarities between MS and SCZ are summarized in this table.

Entry	SCZ	MS
gender different	more males	more females
MHC—complementary associated MHC loci (positive associations)		HLA-DRB1*1501, HLA-DRB1*13:03, HLA-DRB1*03:01, and HLA-DQB1*02:01 alleles
protective MHC loci (negative associations)	HLA-B*08:01, HLA-C*07:01, HLA-DRB1*03:01, HLA-DQA1*05:01, and HLA-DQB1*02:01	HLA-A*02:01
autoantibodies, if present	directed against myelin	directed against receptors
main neurological system	cognition, perception, and affect	sensory and motor systems
main neurological problem	productive information additional information	processing of information

*HLA alleles according to the new 2010 *HLA* Nomenclature (hla.alleles.org/nomenclature/index.html).
